# Supplementary material for: On-chip temporal focusing of elastic waves in a phononic crystal waveguide
Source: Nat Commun. 2018 Apr 6;9:1331. doi: 10.1038/s41467-018-03726-7 (PMC5889409; doi:10.1038/s41467-018-03726-7)
Supplement: Supplementary file 1 — Supplementary Information(PDF 1015 kb) [file 41467_2018_3726_MOESM1_ESM.pdf]

Supplementary Information

On-chip temporal focusing of elastic waves in a phononic crystal waveguide

Supplementary Note 1: Effect of third-order dispersion

The GVD effect, which is proportional to k_2, determines the ultrasonic pulse waveform. However, we also need to take account of the third-order dispersion (TOD) effect $k_3 = \frac{\partial^3 k}{\partial \omega^3}$ when the centre frequency of the pulse approaches the band edges (ref. 1). To evaluate the contribution of these effects, it is useful to introduce dispersion length scales for GVD and TOD (ref. 2),

$$x_{\rm D} = \frac{T_0^2}{|k_2|}, \quad x'_{\rm D} = \frac{T_0^3}{|k_3|}, \tag{1}$$

Using equation (1), equation (6) in the main text can be modified to,

$$\mathrm{i}\frac{\partial U}{\partial x} = \frac{k_2}{2}\frac{\partial^2 U}{\partial T^2} + \frac{\mathrm{i}k_3}{6}\frac{\partial^3 U}{\partial T^3} = \frac{\mathrm{sgn}(k_2)T_0^2}{2x_{\rm D}}\frac{\partial^2 U}{\partial T^2} + \mathrm{i}\frac{\mathrm{sgn}(k_3)T_0^3}{6x'_{\rm D}}\frac{\partial^3 U}{\partial T^3} \tag{2}$$

where $\mathrm{sgn}(k_{i=2,3}) = \pm 1$ depending on the signs of k_2 and k_3. Equation (2) indicates that the GVD effect is dominant in pulse evolution when $x_{\rm D}$ is smaller than $x'_{\rm D}$, but the TOD effect also becomes dominant when $x'_{\rm D}$ approaches $x_{\rm D}$. Supplementary Figure 1(a)-(c) show the frequency dependence of $x_{\rm D}, x'_{\rm D}$ and the ratio $x'_{\rm D}/x_{\rm D}$ of 1D PnC WG, respectively, which are calculated from an FEM simulation. These results indicate that the pulse evolution is mainly dominated by the GVD effect around the centre of the band, where $x'_{\rm D}/x_{\rm D}$ is sufficiently large, and thus, the temporal focusing of the ultrasonic wave at 5.8, 5.35 and 4.5 MHz can be described solely by the GVD effect as shown in Fig. 4(b), Supplementary Figure 2(a) and 2(b), respectively. On the other hand, $x'_{\rm D}/x_{\rm D}$ is small at the band edges, which allows the TOD effect to distort the pulse waveform so that it becomes asymmetric and an oscillatory structure appears near the tailing edge as shown in Supplementary Figure 3(a), and thus the dynamics of the temporal focusing deviates from the GVD theory as shown in Supplementary Figure 3(b).

Supplementary Note 2: Spatial focusing

We evaluate a spatial width of a Gaussian pulse in this section. The pulses are focused in temporal domain, which indicates pulses are also focused in spatial domain. Although a waveform is Gaussian in temporal domain, a waveform is not Gaussian but asymmetry due to group velocity dispersion The spatial full width at half maximum (FWHM) (S_{FWHM}) is evaluated by using the equation (3) (ref. 2).

$$U(x,t) = \frac{T_0}{[T_0^2 - \mathrm{i}k_2 x(1+\mathrm{i}C)]^{1/2}} \exp\left(-\frac{(1+\mathrm{i}C)(t - x/v_{\mathrm{g}})^2}{2[T_0^2 - \mathrm{i}k_2 x(1+\mathrm{i}C)]} \right). \tag{3}$$

v_{g} is the group velocity of center frequency of a pulse. We show spatial information in the most focused case at $C = 9.7$. A pulse width is slightly different from theoretical one (see Fig. 4b in the main text). Therefore we estimate the spatial width of measured value from same temporal width of theoretical one. For example, input pulse is short compared to theoretical one so that we estimate the spatial width from a pulse with same temporal width which propagates 0.6 mm. Supplementary Figure 4(a) and (b) show the normalized spatial waveform which correspond to leftmost and center pulse in Fig. 4b. This indicates that time lens enables S_{FWHM} to be spatially compressed from 0.48 mm to 0.14 mm at $C = 9.7$. If the device can sustain broader band with, the ability to amplify waves is improved.

Supplementary Note 3: Interdigital transducer electrodes

To induce nonlinear effects, interdigital transducer (IDT) electrodes are introduced at the edge of the WG where 50 IDT fingers are arrayed with a pitch of 20 µm as shown in Supplementary Figure 5. The entire length of the WG is 33 mm, and it consists of three 10 mm-long linear parts and two 1.5 mm-long curved parts. Supplementary Figure 5(a) shows an optical microscope image of the IDT electrodes, which enable high intensity ultrasonic waves to be excited by applying an alternating voltage to them as shown in Supplementary Figure 5(c). The WG has periodically arrayed air holes with a pitch of 8 µm that can be used to suspend the membrane with a width of 27.5 µm.

The corresponding dispersion relation, phase velocity and group velocity, GVD coefficients are calculated from an FEM simulation and are shown in Supplementary Figure 5(a)-(c).

Supplementary Note 4: Power flow in the 1D PnC WG

We consider a general Lagrangian for an infinitely long beam as a function of z, $\dot{z} \equiv \partial z/\partial t$, $z' \equiv \partial z/\partial x$, $z'' \equiv \partial^2 z/\partial x^2$, where $z(x,t)$ is the beam displacement, in the form of

$$\mathcal{L} = \int_{-\infty}^{\infty} dx \Lambda(z, z', z'', \dot{z}) \tag{4}$$

where Λ is Lagrangian density. The action is given by

$$S = \int_{t1}^{t2} dt \mathcal{L} = \int_{t1}^{t2} dt \int_{-\infty}^{\infty} dx \Lambda(z, z', z'', \dot{z}) \tag{5}$$

so that its variation is given by

$$\begin{aligned} \delta S &= \int_{t1}^{t2} dt \int_{-\infty}^{\infty} dx \Big[\frac{\partial \Lambda}{\partial z} \delta z + \frac{\partial \Lambda}{\partial z'} \delta z' + \frac{\partial \Lambda}{\partial z''} \delta z'' + \frac{\partial \Lambda}{\partial \dot{z}} \delta \dot{z} \Big] \\ &= \int_{t1}^{t2} dt \int_{-\infty}^{\infty} dx \Big[\frac{\partial \Lambda}{\partial z} - \frac{\partial}{\partial x} \frac{\partial \Lambda}{\partial z'} + \frac{\partial^2}{\partial x^2} \frac{\partial \Lambda}{\partial z''} - \frac{\partial}{\partial t} \frac{\partial \Lambda}{\partial \dot{z}} \Big] \delta z \end{aligned} \tag{6}$$

Here, we performed a partial integration taking into account the boundary condition whereby the displacement vanishes at a sufficiently long distance. To satisfy the least action principle $\delta S = 0$ for any variation δz, the following Lagrange field equation should be satisfied

$$\frac{\partial \Lambda}{\partial z} - \frac{\partial}{\partial x} \frac{\partial \Lambda}{\partial z'} + \frac{\partial^2}{\partial x^2} \frac{\partial \Lambda}{\partial z''} - \frac{\partial}{\partial t} \frac{\partial \Lambda}{\partial \dot{z}} = 0. \tag{7}$$

If we define $h(x,t)$ and $s(x,t)$ as

$$h(x,t) = \dot{z} \frac{\partial \Lambda}{\partial \dot{z}} - \Lambda, \; s(x,t) = \frac{\partial \Lambda}{\partial z'} \dot{z} + \frac{\partial \Lambda}{\partial z''} \overleftrightarrow{\partial}_x \dot{z}, \quad \left(A \overleftrightarrow{\partial}_x B \equiv A \frac{\partial B}{\partial x} - \frac{\partial A}{\partial x} B \right), \tag{8}$$

we can easily derive a one-dimensional continuity equation by using the Lagrange equation as

$$\frac{\partial h(x,t)}{\partial t} + \frac{\partial s(x,t)}{\partial x} = 0. \tag{9}$$

Because $h(c)$ is the Hamiltonian density, this equation expresses the energy conservation law so that $s(x,t)$ gives a generalized form of the energy flow density. With an Euler Bernoulli system, Λ does not include z' and the Lagrangian density and the field equation, respectively, are given by

$$\Lambda = \frac{\rho A}{2}\dot{z}^2 - \frac{EI_y}{2}z''^2, \quad -EI_y\frac{\partial^4 z}{\partial x^4} - \rho A\ddot{z} = 0 \tag{10}$$

The second is the well-known Euler Bernoulli equation. $h(x,t)$ and $s(x,t)$ are then

$$h(x,t) = \frac{\rho A}{2}\dot{z}^2 + \frac{EI_y}{2}z''^2, \quad s(x,t) = -EI_y z''\overset{\leftrightarrow}{\partial}_x\dot{z} \tag{11}$$

The detailed forms of h and s for a plane wave $z(x,t) = z_0\cos(kx-\omega t)$ are given by

$$h(x,t) = \frac{\rho A}{2}\dot{z}^2 + \frac{EI_y}{2}z''^2 = \frac{\rho A\omega^2 z_0^2}{2} \tag{12}$$

$$\begin{aligned} s(x,t) &= -EI_y z''\overset{\leftrightarrow}{\partial}_x\dot{z} = \omega k^3 EI_y z_0^2 \\ &= (\omega/k)\omega^2\rho A z_0^2 = v_{\rm g}h = 2v_{\rm ph}h \end{aligned} \tag{13}$$

Here we used the dispersion relation $\omega = k^2\sqrt{EI_y/\rho A}$ satisfied for the Euler Bernoulli system.

We calculate the power flow due to the pump vibration in a WG by using equation (13). We use $\rho = 5.32\times10^3$ kg m^{-3}, $A = 5.5\times10^{-12}$ m^2, $\omega = 2\pi\times5.5112$ MHz, $v_{\rm g} = 128.9$ m s^{-1}, and a wave displacement of 1 nm. From these values, we calculate the power flow resulting from the pump vibration $P_{\rm p} = 2.3\times10^{-9}$ W .

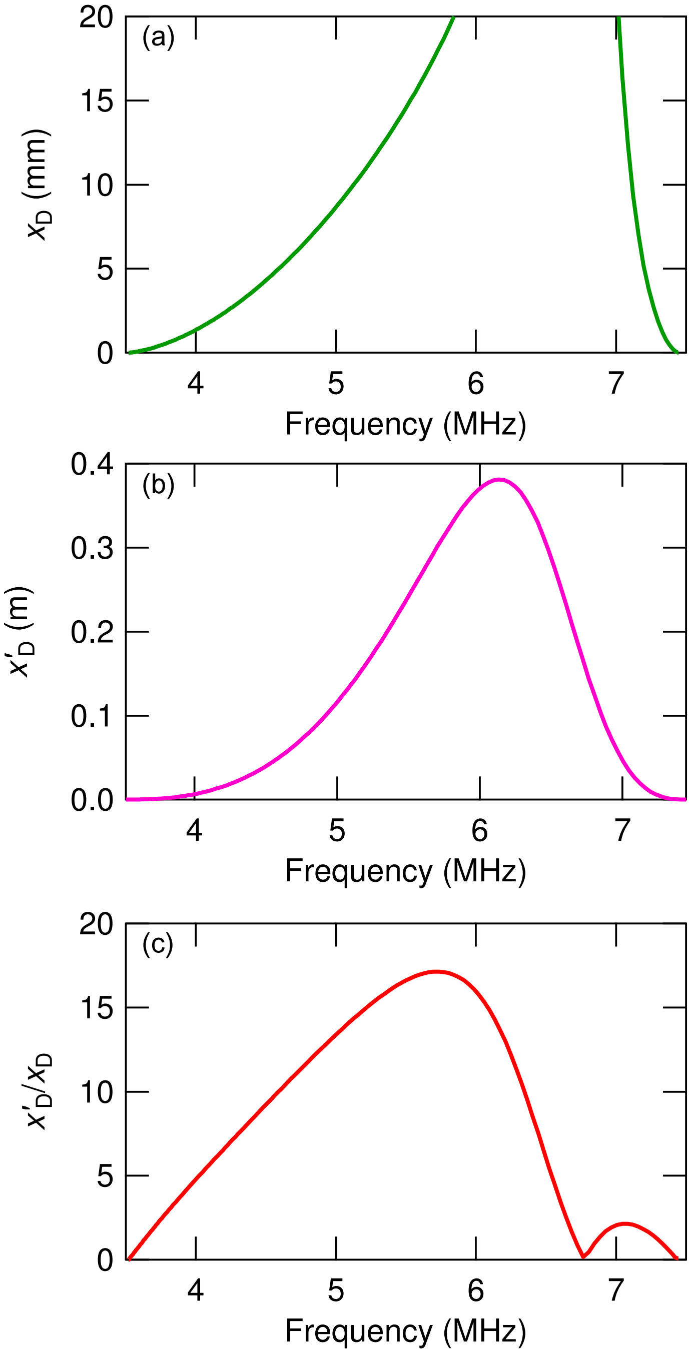

Supplementary Figure 1: **Higher order dispersion length.** (**a**)-(**c**) The frequency dependence of x_{D}, x'_{D} and the ratio of x'_{D} to x_{D}, respectively. These values are calculated from equation (1) using $T_0 = 2.5$ µs.

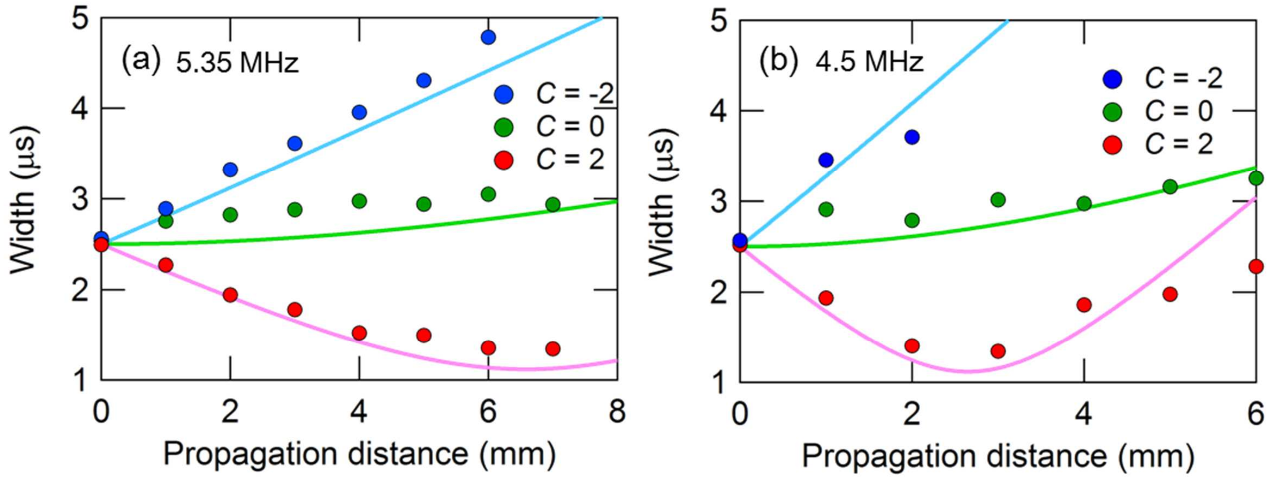

Supplementary Figure 2: **Second order dispersion effect.** (**a**), (**b**) Temporal pulse width as a function of propagation distances when exciting a 2.5 μs chirped pulse with centre frequencies of 5.35 and 4.50 MHz with chirp parameters of ±2 and 0, respectively. Solid lines indicate the theoretical results when $x'_{\mathrm{D}}/x_{\mathrm{D}} = 15.8$ (a) and 9.2 (b).

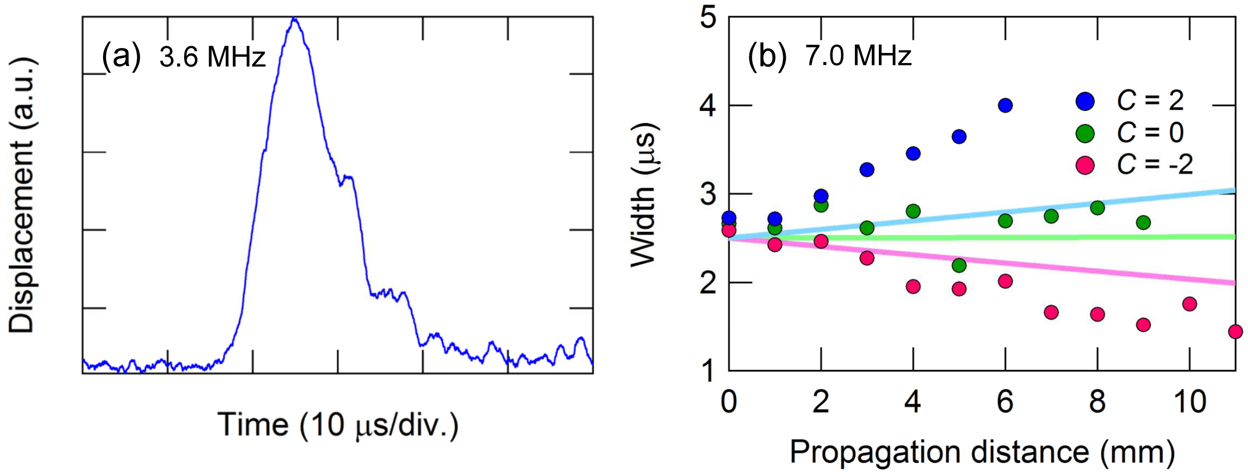

Supplementary Figure 3: **Third-order dispersion effect.** (**a**) Temporal response of an ultrasonic wave realized by exciting an unchirped Gaussian input pulse with $T_0 = 1.2$ μs when $x'_{\mathrm{D}}/x_{\mathrm{D}} = 0.8$. (**b**) Temporal pulse width as a function of propagation distance when exciting a 2.5 μs chirped pulse with a centre frequency of 5.8 MHz with chirp parameters of ±2 and 0. Solid lines indicate the theoretical results when $x'_{\mathrm{D}}/x_{\mathrm{D}} = 2.1$.

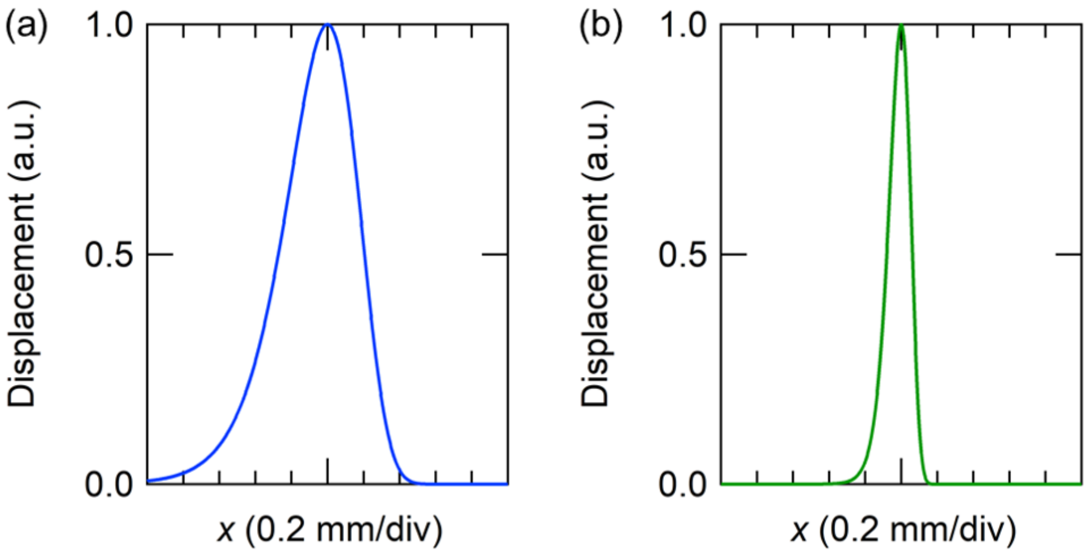

Supplementary Figure 4: **Spatial pulse waveform.** (**a**) Input pulse waveform. (**b**) Most compressed pulse waveform.

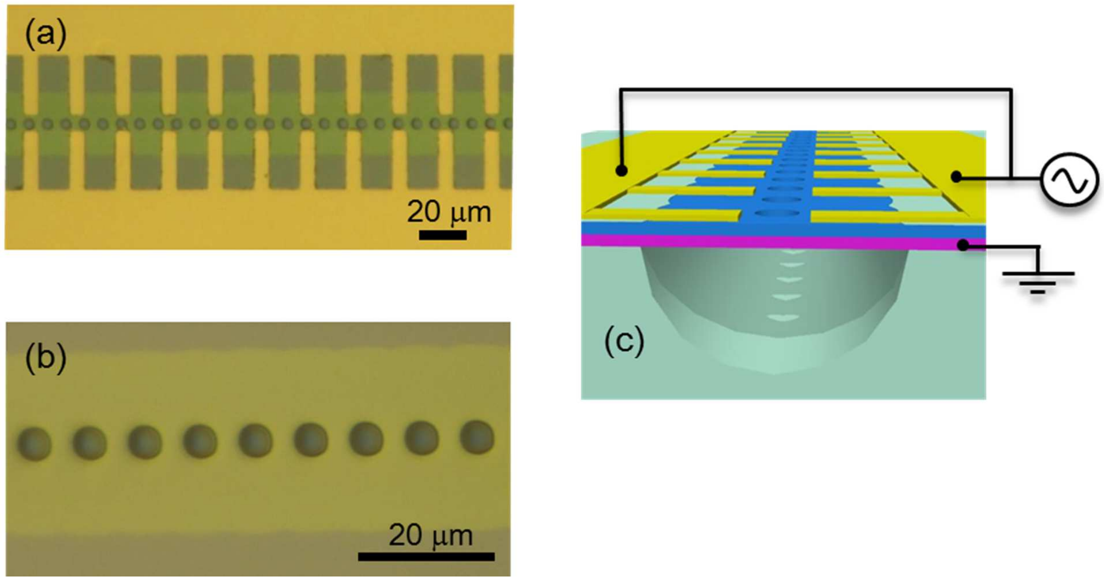

Supplementary Figure 5: **1D PnC WG with IDT electrodes.** (**a**) Optical micrograph of a 1D PnC WG where IDT electrodes (yellow) are formed on the suspended membrane (green). (**b**) Optical micrograph of the suspended membrane. (**c**) A schematic of the device cross-section where an electric field is applied perpendicular to the out-of-plane direction.

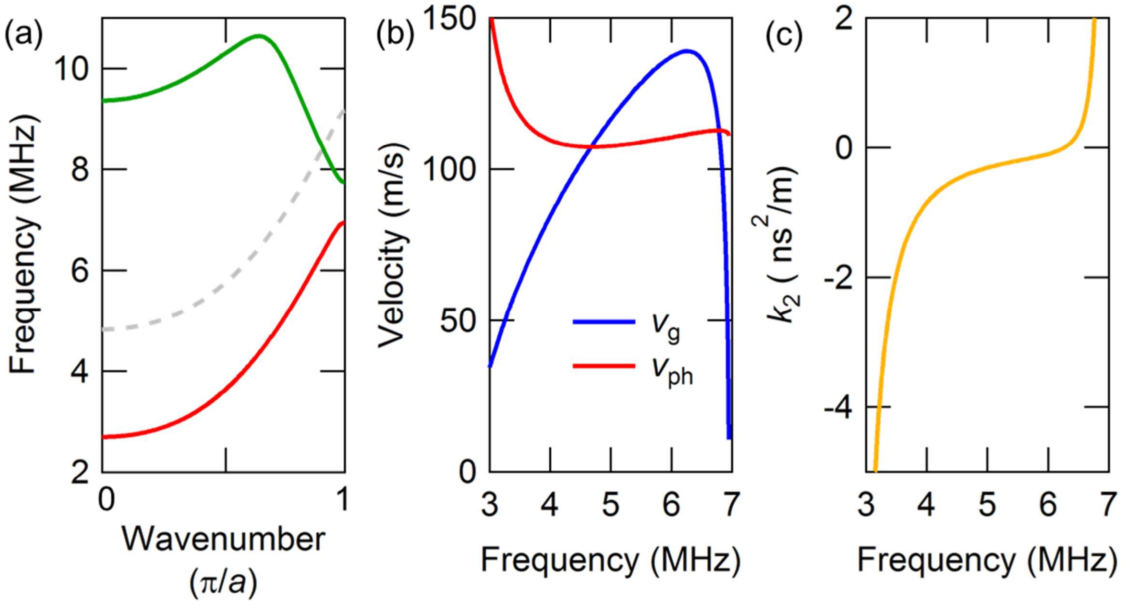

Supplementary Figure 6: **Fundamental properties of the PnC WG with IDT electrodes.** (**a**) The FEM simulated dispersion relation. (**b**) The frequency dependence of the group velocity (v_{g}) and the phase velocity (v_{ph}). (**c**) The frequency dependence of GVD coefficient k_2.

Supplementary References

1. Marcuse, D. Pulse distortion in single-mode fibers. 3: Chirped pulses. *Appl.Optics* **20**, 3573 (1981).

2. Agrawal, G. *NonlinearFiberOptics*. Boston, fifth edition, (2013).
